# The Making of New Features

**Published:** 1889-04-20

**Authors:** 


### THE MAKING OF NEW FEATURES.

One of the most interesting of the many new operations practised by modern surgeons is the transplantation of portions of skin from one part of the body to another. A piece of healthy skin may be cut away, say, from the arm or other place where a slight disfigurement is unimportant, and if properly prepared may be applied to a part that has been denuded of skin by disease or otherwise, and it will in most cases attach itself by vital union to its new resting-place, and become to all intents and purposes a "new feature." For example, new eyelids are produced in this way, new wings to noses, and so on. Dr. Wolfe, of Glasgow, reports several cases, on the authority of Prof. von Esmarch, which well illustrate this operation. The first is that of a medical student. This embryo anatomist, instead of confining his carving of the human body to the subjects of the dissecting-room, had a fancy for duelling. He engaged in a duel with swords in 1882, and received from his opponent's weapon a deep wound in the bridge of the nose. From the left wing of that organ also a piece as large as a florin was completely cut away, leaving a hideous hole. The under half of the nose was deeply gashed, and hanging downwards. If ever a feature was destroyed, surely this medical student's nose was that feature. But the surgeon in charge by no means despaired. The hanging-down portion was speedily stitched with catgut into its proper place. A portion of skin large enough to fill the "florin hole" was taken from the forearm, and fixed by compresses to the place it was designed to occupy; and the wound thus treated, with antiseptic precautions, was left bandaged for five days. The compress was then removed, and the transplanted piece of skin was found everywhere grown to its new bed in the nose, whilst that organ was restored to a high degree of respectability of appearance. But for this operation, that medical student's face would have been too repulsive for him to have ever thought of practising as a physician or surgeon.

### Madame K.'s Nose.

Medical men will appreciate still more the following important case. Madame K., the wife of a lieutenant-colonel, had suffered for seven years before 1876 from a rodent ulcer of the nose. A rodent ulcer, as its name implies, is a spreading sore of a kind very difficult to cure, and very liable to recur. The history of the case well shows both the persistent character of a rodent ulcer and the great value of a permanent cure. The ulcer, when first seen by a surgeon, was not larger than a grain of seed. Its situation was the bridge of the nose, and it was both painful and disfiguring. After a time the nodule fell out, and left a hole, as Dr. Wolfe, with more regard for literalness than for the nerves of sensitive readers, says, "like that of a maggot." In 1878 the disease had returned. In the following year it was removed, and for a time the wound healed. Two years later, the affected part had become more diseased than before. There were then two red cicatrices, each as large as a coffee-bean, and each covered with an ugly scab. Again treatment was resorted to. A powerful caustic was applied, and remedies were given internally with a view of improving the constitutional condition. Once more the parts healed, and hopes of permanent recovery again began to be entertained. But these hopes proved to be as futile as before. In no long time the pains became very severe, and the wound, which had been closed for some time, once more reopened. Then the patient did what patients under such circumstances so very often and so very naturally do—she went about from surgeon to surgeon, from allopath to homœopath, and from one homœopath to another. But not one of them, either orthodox or heterodox, succeeded in effecting a cure. The disease steadily grew worse, and the patient gave way to despair. On March 14, 1883, seven years from its commencement, the ulcer had taken on a new character, and a "great part of the bridge of the nose was covered with cancer." It was then resolved to remove the diseased portion entirely, and to try the effect of transplanting a flap of skin from the left arm. This was done, and the part of the nose left bare by the removal of the cancer and a little of the healthy tissue surrounding it, was accurately fitted with the new and healthy skin. It was necessary to stitch the skin to the nose, and, as before, to use antiseptic applications to prevent suppuration. The result was admirable. In five days the flap of skin had become perfectly united to the nose, and all sign of disease had disappeared. The best remains to be told. It is now five years since the date of the operation, and not only has there been no return of the disease, but the new skin of the nose is almost undistinguishable from that of the surrounding parts. In Madame K.'s case orthodox medicine undoubtedly carried off the honours.

